# Signaling pathways in cutaneous wound healing

**DOI:** 10.3389/fphys.2022.1030851

**Published:** 2022-11-25

**Authors:** Olga Krizanova, Adela Penesova, Jozef Sokol, Alica Hokynkova, Amir Samadian, Petr Babula

**Affiliations:** ^1^ Institute of Clinical and Translational Research, Biomedical Research Center SAS, Bratislava, Slovakia; ^2^ Department of Chemistry, Faculty of Natural Sciences, University of St. Cyril and Methodius, Trnava, Slovakia; ^3^ Department of Physiology, Faculty of Medicine, Masaryk University, Brno, Czechia; ^4^ Department of Burns and Plastic Surgery, Faculty of Medicine, Masaryk University and University Hospital, Brno, Czechia

**Keywords:** wound healing, gasotransmitters, calcium, hydrogen peroxide, diabetes mellitus

## Abstract

Wound healing is a very complex process, where variety of different pathways is activated, depending on the phase of healing. Improper or interrupted healing might result in development of chronic wounds. Therefore, novel approaches based on detailed knowledge of signalling pathways that are activated during acute or chronic cutaneous wound healing enables quicker and more effective healing. This review outlined new possibilities of cutaneous wound healing by modulation of some signalling molecules, e.g., gasotransmitters, or calcium. Special focus is given to gasotransmitters, since these bioactive signalling molecules that can freely diffuse into the cell and exert antioxidative effects. Calcium is an important booster of immune system and it can significantly contribute to healing process. Special interest is given to chronic wounds caused by diabetes mellitus and overcoming problems with the inflammation.

## Introduction

Skin as the biggest organ in humans provides several important functions for the organism—it acts as a barrier maintaining skin integrity and homeostasis against harmful pathogens and physical stressors. Acute (mechanical injury, surgery, burn, etc.) or chronic (diabetic ulcers, etc.) cutaneous damage can have serious consequences to the whole body. Therefore, wound healing as a multistep process is an important move in the maintenance of human health and well-being. Hemostasis, inflammation, proliferation, and remodelling belong to the main steps in wound healing (Borena et al., 2015).

Skin consists of two layers—thin epithelial membrane (epidermis) and a thicker layer (dermis), composed of connective tissue. These layers differ in the composition and also in the function. Various types of cells can be recognized in both layers. In dermis, six cell types that differently contribute to wound healing were identified. Also, myofibroblasts and macrophages may change the skin wound healing fates by modulating critical signalling pathways (Chen et al., 2022). Single cell analysis revealed heterogeneity in large wounds (Guerrero-Juarez et al., 2019). In murine skin wounds the dynamic nature of fibroblast identities was shown during healing with formation subclusters of the wound fibroblasts into distinct cell populations. Also, the wound induced plasticity of myeloid lineage cells was demonstrated on this model (Guerrero-Juarez et al., 2019). Major variations in epithelial, fibroblast, and immune cell populations were observed in young and aged skin during wound healing (Vu et al., 2022). It is well known that wound healing declines with age, which contributes to a variety of health complications, and to decreased lifespan. Aged skin wounds exhibited more inflammatory profile than young equivalents, probably due to dysregulated growth factor, chemokine, and cytokine pathways during wound healing in aged skin (Vu et al., 2022). Moreover, aged basal epidermal keratinocytes isolated from the wound edge appeared to be more recalcitrant to activation, as judged by their markedly reduced transcriptional activity of genes involved in important processes of wound-repair (Keyes et al., 2016).

Wound healing is a complex process that involves the interaction between different cell types, growth hormones, cytokines, antioxidants and a stable supply of metal ions (e.g., calcium, zinc, and magnesium) (Dehkordi et al., 2019). After the skin is damaged, several cell systems and signalling pathways are activated in the wound to defend the body. Therefore, and also due to complexity of the skin, diverse approaches are needed to improve cutaneous wound healing (Zeng et al., 2018). Due to differences in signalling, healing strategy of acute and chronic wounds diverge. While acute wound heals in 3–4 weeks depending on the size, localization, origin, patient’s co-morbidities, age, etc., chronic wound basically stops in the certain phase of healing, generally in the inflammatory phase. Also, chronic wounds are characterized by persistent infections, formation of drug-resistant microbial biofilms and the inability of dermal and/or epidermal cells to respond to reparative stimuli (Table 1, Demidova-Rice et al., 2012). Besides inflammatory phase, basic differences between acute and chronic wounds occur also in proliferative phase (Martin and Nunan, 2015). In the acute wounds platelets release platelet-derived growth factor and transforming growth factors A1 and 2, which attract inflammatory cells that release reactive oxygen species (ROS) and effectively clear the wound from bacteria (Demidova-Rice et al., 2012). Afterwards, growth factors are produced to induce and maintain cellular proliferation while initiating cellular migration. Finally, granulation tissue is formed to support epithelialization (Demidova-Rice et al., 2012). In chronic wounds, lower density of growth factor receptors occur that decrease the mitogenic potential of dermis and epidermis. Keratinocytes derived from chronic ulcers have increased expression of several cell cycle–associated genes, such as cyclin-dependent protein kinase 2 and cyclin B1, which point to the hyperproliferative status. However, these chronic wound–derived keratinocytes with increased proliferative marker Ki67 exhibit impaired migratory potential (Demidova-Rice et al., 2012; Martin and Nunan, 2015). Therefore, chronic wounds caused by progression of some diseases (e.g., diabetes) require repetitive or periodical medical intervention to prevent complications.

**TABLE 1:** Major differences between acute and chronic wounds.

Acute wounds	Chronic wounds
Time of healing is 3–4 weeks	Time of healing is long, or wounds are nontreatable
Activation of resident immune cells	Persistent inflammation and formation of drug-resistant microbial biofilms
Release of cytokines	Alterations in inflammatory cytokines
Stimulation of fibroblasts	Fibroblast senescence
Deposition of extracellular matrix	Decreased extracellular matrix
Neovascularization, angiogenesis	Impaired angiogenesis

**FIGURE 1:**
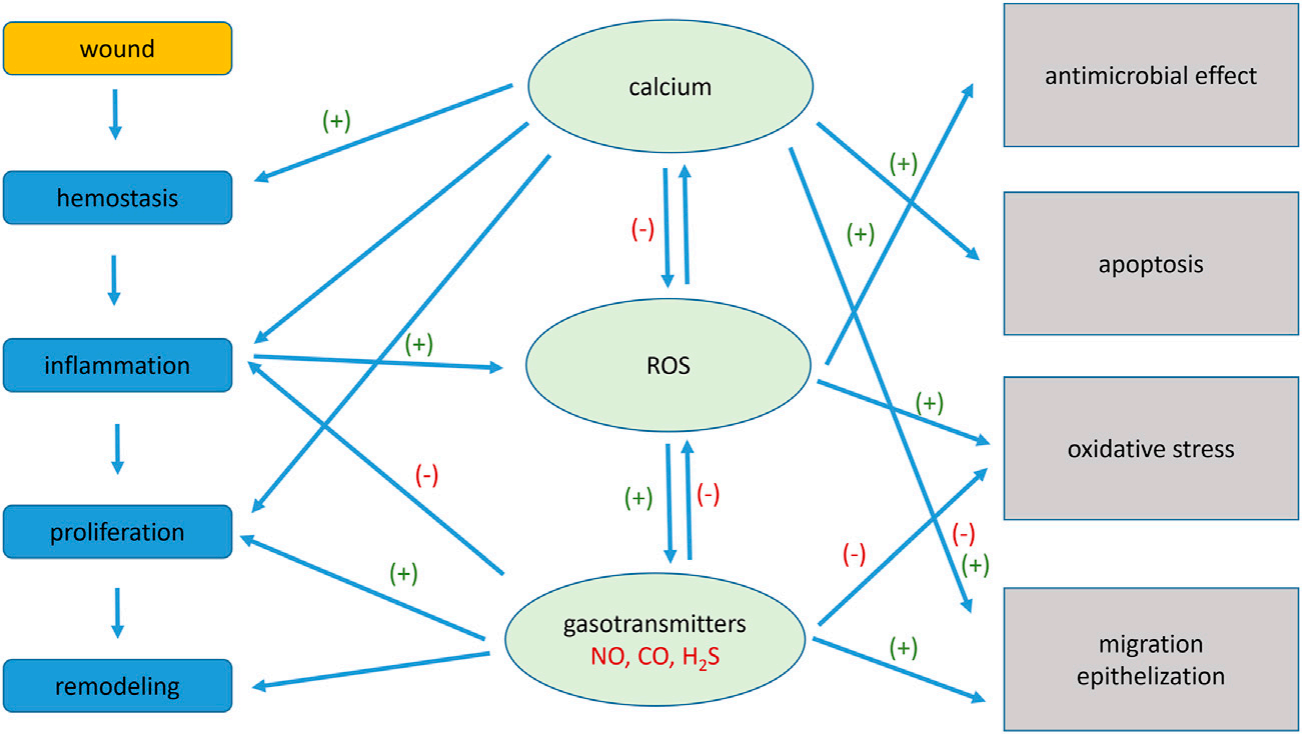
Schematic interaction of signalling molecules (calcium ions, ROS and gasotransmitters) in the individual phases of wound healing and their effects. Calcium ions affect hemostasis, inflammation and also proliferative phase of wound healing, which have an impact on the process of apoptosis, epithelization and migration. Gasotransmitters affect inflammation, proliferation and remodelling and significantly affect the amount of reactive oxygen species (ROS) and thus oxidative stress and also epithelization and migration. (+) represents positive effect, (−) represents negative effect.

Inflammation is the basic response to cutaneous wounds that helps to protect the tissue from further damage and set up conditions that promote repair. Inflammation as the early step of the wound healing is characterized by the overproduction of ROS. Although the precise role of ROS in the process of wound healing is still not fully clear, increasing evidence suggests that ROS might be crucial for wound repair, not only as germicides but also for cellular signalling (Roy et al., 2006) in different phases of wound healing (for review see André-Lévigne et al., 2017). To eliminate excessive ROS production antioxidants and/or gasotransmitters are among the interest in many fields of medicine. Gasotransmitters are signalling molecules that easily penetrate through the plasma membrane and they have well defined and specific functions at physiologically relevant concentrations (Shefa et al., 2017). Exogenous application of gasotransmitters to wounds can significantly improve their treatment. Calcium ions play an unmistakable role in wound healing. It was proved that dietary calcium deficiency caused delayed wound healing and higher prevalence of chronic wound formation (Lansdown, 2002). Recently, photothermal injectable hydrogel composed of Ca^2+^ and alginate solution with α-lipoic acid modified palladium nanoparticles was developed, and possess anti-oxidative and anti-inflammatory properties (Luo et al., 2022). Role of the calcium ions in healing process is well documented not only during inflammation, but also in the proliferation phase (Subramaniam et al., 2021). Thus, new approaches based on calcium therapy (and combined calcium and vitamin D therapy) can result in more effective wound healing. Also, ROS can affect calcium signalling through targeting its influx through calcium channels (Gorlach et al., 2015). Mutual communication of calcium signalling, ROS and gasotransmitters is shown in Figure 1.

This review is focused on possibilities to utilize gasotransmitters and calcium ions and/or their combination in wound healing under normal and special conditions (diabetes). We focus on wound healing in diabetes as a modern civilization burden, which significantly contributes to chronic wound healing problems.

## Wound healing, reactive oxygen species, and gasotransmitters

Increased reactive species (ROS) production serves as a defence to fight against pathogen attacks. Thus, ROS accumulation is required to prevent infection in the area of the wound (Muzumdar et al., 2019). However, long-term exposure to high concentrations of ROS generally causes oxidative stress, which damages cells (Figure 1). ROS contributes to the increasing group of gaseous mediators in the control of wound healing. Inhibiting excessive ROS production is an important feature in wound healing. From this point, antioxidants might play an important role in this process. Nrf2-activating compounds were studied to prevent and treat chronic inflammatory and degenerative disorders. It has been shown that Nrf2-inducing bioactive compounds that improve the wound healing process may be a promising therapeutic approach for treating chronic wounds (Suntar et al., 2021). Li and co-workers have shown that a hydrogen-rich medium relieved oxidative stress *via* activation of the Nrf-2/heme oxygenase-1 (HO-1) pathway (Li et al., 2022). Also, gasotransmitters serve as a barrier to increased ROS, particularly to superoxide radicals. To promote gas-healing therapy, the following requirements should be fulfilled: 1) biocompatibility, 2) ability to provide adequate and controlled amounts of gasotransmitter, 3) protection of the wound against pathogens, and 4) retaining a favourable moist wound environment (Schneider et al., 2009). Three gasotransmitters, nitric oxide (NO), carbon monoxide (CO), and hydrogen sulfide (H_2_S) are important players in wound healing. These gasotransmitters are endogenously produced, but they could be donated also exogenously.

Nitric oxide, the best-described gasotransmitter, is deeply involved in the modulation of a variety of cellular functions, especially in the heart and nervous system (Förstermann and Sessa, 2012). It can be produced endogenously by three types of NO synthases (NOS)—neuronal (nNOS), endothelial (eNOS), inducible (iNOS), or added to the cells exogenously. NO was also described to affect cutaneous functions, like proliferation, differentiation, or keratinocyte migration (Krischel et al., 1998; Zhan et al., 2015). NO seems to have a biphasic effect on wound healing. Low levels of NO can increase the permeability of the endothelium and facilitate the migration of inflammatory cells to the affected site, thus positively affecting cytokine expression. On the other side, high levels of NO can inhibit cutaneous inflammation, probably by inhibiting migration and adhesion of inflammatory cells (Man et al., 2022a). In wounds, NO is generated mainly by iNOS. The iNOS plays an essential role in non-specific defence against microorganisms (Man et al., 2022b). It was shown that iNOS-deficient mice showed a severely delayed epithelial wound closure (Yamasaki et al., 1998). Also, NO can sensitize and enhance the antibacterial effectiveness of many therapeutic approaches, such as antibiotics. To provide an adequate and accurate amount of NO to the wound, several new approaches were tested and developed. Polymer matrices are of special interest for NO molecular systems functionalization due to their high versatility and similarities with living tissues (for review see work of Pinto et al., 2022). Recently, NO-releasing oxidized bacterial cellulose/chitosan crosslinked hydrogel was shown to eliminate polymicrobial wound infection, where linear polyethyleneimine diazeniumdiolate was used as the NO donor (Hasan et al., 2022). This newly developed NO-releasing hydrogel represents a promising approach for the treatment of various skin infections. Another approach utilizes a type of gold nanostar/hollow polydopamine Janus nanostructure with precise near-infrared - controlled NO release property, which effectively eliminated methicillin-resistant *Staphylococcus aureus* from infected wounds and promoted wound healing through a synergistic photothermal and NO therapeutic effect (Liang et al., 2022).

Carbon monoxide (CO) is endogenously produced by heme oxygenases (HO) and its beneficial effect is also dependent on its concentration. Three isoforms of this enzyme were described up to now, inducible type HO-1, and constitutively expressed types HO-2 and HO-3. HO-1 protects against oxidative stress and is regulated by the redox-sensitive transcription factor, the nuclear factor (erythroid-derived 2)-like 2 (Nrf2). Abundantly produced CO in activated macrophages can enhance proliferation, differentiation, and polarization towards anti-inflammatory effects on cells (Kang et al., 2021). Vectorization of CO releasing molecules by gold nanoparticles was shown to improve the anti-inflammatory effect of CO (Fernandes et al., 2020). Recently, a new strategy of the activation of CO-release from 3-hydroxyflavone moieties through a photooxygenation mechanism was described, thus enabling CO to release under red light irradiation, exerting a selective antimicrobial effect on *S. aureus* bacteria (Cheng et al., 2021). Combined and simultaneous release of NO and CO from a single donor molecule (obtained by covalent grafting of NO-releasing N-nitrosamine onto the CO-releasing 3-hydroxyflavone derivatives under visible light irradiation) exerted a synergistic antibacterial effect against *S. aureus* (Gao et al., 2022). Nevertheless, application of an exogenous CO might be a problem, since it is difficult to quantify precise amount of administered CO and its administration might increase plasma carboxyhemoglobin to toxic levels (Takagi et al., 2022).

The third gasotransmitter—hydrogen sulfide (H_2_S)—was described to affect a variety of body functions, including cardiovascular, neurological, reproductive, and endocrine systems. Also, it was shown to affect the cancer proliferation, but also apoptosis, since its effect is bell-shaped (Cao et al., 2019; Kajsik et al., 2022). H_2_S is also involved in wound healing, mainly because of the anti-inflammatory properties and attenuation of oxidative-stress-related tissue injury (Figure 1). Mechanism of the beneficial effect on exogenous supplementation can cover also vascular endothelial growth factor upregulation, which might promote blood vessel formation, increase blood perfusion around the wound, and finally accelerates wound healing (Xu et al., 2019). Kutz and co-workers have found that H_2_S mediates cutaneous vasodilation and has a functional interaction with both NO and cyclooxygenase signalling pathways (Kutz et al., 2015). H_2_S is produced endogenously by three enzymes—cystathionine β-synthase (CBS), cystathionine γ-lyase (CSE), and 3-mercaptopyruvate-sulfurtransferase (MST). CSE appeared to be the most relevant H_2_S-producing enzyme in wound tissue (Goren et al., 2019). Wu and co-workers developed a novel PCL fibrous matrix coated with pH-controllable H_2_S releasing donor JK1 (Wu et al., 2016). This matrix promoted wound healing efficiency through H_2_S’s unique cytoprotective characteristics *in vivo*. Other carriers for JK1 encapsulation—sodium alginate or a hyaluronic acid-based hydrogel were also tested (Chattopadhyay et al., 2016; Zhao et al., 2020). Up to now, the controversial effect of the H_2_S in inflammation caused by burns was described. The effect of H_2_S might depend on the extent of burn degree, the course of the burn, or dosage of H_2_S, and the treatment time with H_2_S donors. Due to the biphasic effects of H_2_S on burn wounds, H_2_S supplementation in the late, but not the early stage of a burn may be helpful to accelerate healing (Xu et al., 2021).

Hydrogen peroxide (H_2_O_2_) is an endogenous reactive oxygen species that contributes to oxidative stress directly as a molecular oxidant and indirectly through free radical generation. It has antimicrobial properties and can act as a debriding agent through its effervescence, making low-concentration H_2_O_2_ useful for wound care. H_2_O_2_ has also been shown to promote venous insufficiency ulcer healing (Murphy and Friedman, 2019). H_2_O_2_ is very important signalling molecule. In the zebrafish animal model, where the wound was induced mechanically, H_2_O_2_ production was detected in the wound margins, with its concentration increasing over time along with leukocyte recruitment with a peak at 20 min. Its formation is mediated predominantly by NADPH oxidase, which converts oxygen to the superoxide anion radical, which is further converted by superoxide dismutase to hydrogen peroxide. NADPH oxidase is activated/stimulated not only by mechanical injury but also by pathogenic microorganisms or pro-inflammatory cytokines (Zhu et al., 2017). In the phase of haemostasis, hydrogen peroxide stimulates the exposure of tissue factor to the surface of the relevant cells involved in haemostasis, initiating a cascade of actions leading to the generation of thrombin, the central molecule of haemostasis. It also affects platelet adhesion and aggregation (Sen and Roy, 2008). In the inflammatory phase, hydrogen peroxide affects the efficiency of macrophages at the level of protease secretion, stimulates the release of pro-inflammatory cytokines and also stimulates the recruitment of additional macrophages. It is involved in neutrophil extracellular trap formation. Hydrogen peroxide as a non-radical form of ROS is able to participate in the formation of microbially or oxidatively more efficient compounds such as hypothiocyanite (Zhu et al., 2017). In some works, H_2_O_2_ has been shown to have the ability to induce TNF-β production and stimulate fibroblast proliferation, leading to increased fibrotization. Here we are already at the level of cell proliferation and remodelling phases. Excessive stimulation of TGF-β leads to accelerated wound healing, but it is accompanied by increased fibrosis and scar formation. Hydrogen peroxide stimulates the production of certain growth factors, such as VEGF, which is released by macrophages and stimulates angiogenesis, and this effect is concentration dependent. In *in vivo* models, ability to affect keratinocyte viability and migration has been demonstrated (Urban et al., 2019). The effect of hydrogen peroxide on the secretion of other physiologically active molecules involved in wound healing remains a question. Here, it would certainly be worth mentioning cyclooxygenase-2 (COX-2), which is crucial for the formation of prostacyclins and prostaglandins. These actions influence a variety of processes including blood flow, vascular tone or angiogenesis. Work by Eligini et al. (2009) showed the ability of hydrogen peroxide to stimulate COX-2, but in endothelial cells. Thus, this area remains virtually unanswered and further studies are necessary.

Use of all mentioned compounds is extremely dependent on the type of dressing. Dressing generally depends on the type of wound, its stage, but also on the type of compound it has to carry. Dressings can be classified from different points, e.g., thein function in the wound healing, type of material, physical form, etc. [for review see (Boateng et al., 2008)]. Modern systems capable of controlled oxygen release are based on oxygen releasing polymeric microspheres [by incorporating hydrogen peroxide into poly (lactic-co-glycolic acid)] and hydrogel scaffolds (Choi et al., 2018), cyanoacrylate-encapsulated calcium peroxide (Zhang et al., 2020), OxOBand composed of antioxidant polyurethane (PUAO), as highly porous cryogels with sustained oxygen releasing properties (Shiekh et al., 2020), oxygenated-bacterial-cellulose nanofibers (Sarkandi et al., 2022), or injectable hydrogel based on hyaluronic acid-graft-dopamine and polydopamine coated Ti3C2 MXene nanosheets (Li et al., 2022). ROS-responsive oxygen and NO releasing systems based on encapsulated biosafe NO donor L-arginine and hydrogen peroxide were developed too (Yu et al., 2022). Other therapeutic approaches include topical application of growth factors and cytokines and some other agents such as hyaluronic acid or erythropoietin. These are also being tested in topical forms, but in controlled-release systems (Legrand and Martino, 2022). Examples include various polymers, particularly modified celluloses, which have the ability to form hydrogels and release growth factors in a controlled manner. Their major advantages include in particular their biocompatibility. Most recently, Hao et al. (2022) have prepared multifunctional benzaldehydeterminated 4-arm PEG (4-arm-PEG-CHO)/carboxymethyl chitosan (CMCS)/basic fibroblast growth factor (bFGF) hydrogels, that have shown the ability to increase Ki67, increase generation of epithelialization and collagen, induces the formation of hair follicles, and enhanced neovascularization by upregulating the production of CD31 and CD34 (Hao et al., 2022). A similar approach was taken by Cheng et al. (2020), who, however, used metal-free CO-releasing polymers based on photoresponsive 3-hydroxyflavone derivatives (Cheng et al., 2020).

## Involvement of calcium channels in wound healing

A variety of ions is indisputably involved in different stages of wound healing. Calcium ions are involved in both, normal skin function and also in wound healing. Calcium ions are prerequisite for keratinocyte differentiation and corneocyte formation. To cope with the different calcium needs of keratinocytes (low calcium concentrations for proliferation, high calcium for differentiation) epidermis built up calcium gradient (Rinnerthaler and Richter, 2018). Calcium can enter the cytoplasm of cells either from outside, through special types of calcium channels, or by release from the intracellular stores, mainly from the endoplasmic reticulum (for review see Babula and Krizanova, 2022). The function of individual calcium transport systems in wound healing is unwinded from their role in healthy skin. For example, ryanodine receptors (RyRs) that are localized in the membranes of the endoplasmic reticulum are expressed in keratinocytes and can affect their differentiation and barrier homeostasis (Denda et al., 2012). After the skin wound creation, the initiation of keratinocyte migration is among the first reparation mechanisms (O’Toole, 2001). In this process, an increase in the intracellular calcium concentration was determined, which probably results in the upregulation of bicarbonate transporter type 2 (AE2). An increase in AE2 expression is probably involved in cell migration and results in wound closure (Hwang et al., 2020). Inhibition of RyRs by specific antagonists (e.g., dantrolene) can accelerate wound closure *in vivo* through the process of epithelialization (Degovics et al., 2019). RyRs are probably activated by exposure to ROS (Csordas and Hajnoczky, 2009). Thus, the limitation of calcium release by inhibition of RyRs resulted in a decrease in ROS formation (Degovics et al., 2019). Based on these results authors have concluded that dantrolene might be another tool for the acceleration of wound healing. The role of other store-operated channels—inositol 1,4,5-trisphosphate receptors (IP_3_Rs)—in wound healing is still elusive. In general, IP_3_Rs type 1 and 2 were shown to have proapoptotic effects in cancer cells, while type 3 IP_3_R has anti-apoptotic effect (Rezuchova et al., 2019). Their importance in wound healing has not yet been fully elucidated. It was already shown that IP_3_Rs activated by phospholipase C are active in human keratinocytes (Tu et al., 2005).

Transient receptor potential (TRP) channels are a diverse group of channels with different function in various tissues. In non-excitable cells, TRP channels regulate intracellular calcium concentrations, which are related to keratinocytes proliferation and differentiation to influence the skin barrier (Moran, 2018). The family of TRP channels comprises a large number of channels that can be divided into 6 subtypes—TRPA (ankyrin), TRPC (canonical), TRPM (melastatin), TRPML (mucolipin), TRPP (polycystin), and TRPV (vanilloid) (Montell et al., 2002). Different TRP channels participate in different skin homeostasis and barrier functions. A variety of TRPC channels was shown to be expressed in keratinocytes and probably playing role in keratinocyte differentiation (Caterina and Pang, 2016). TRPV channels are sensitive to various tissue-damaging signals and their activation is generally perceived as pain (for review see Wang, 2021a). Non-selective ion channel—transient receptor potential vanilloid 1 (TRPV1) is a potential drug target for improving the outcome of inflammatory/fibrogenic wound healing, especially cornea (Nidegawa et al., 2014).

During aging, changes in pH and calcium transport are detectable in skin. The pH of the epidermis goes up and the calcium gradient goes down (Rinnerthaler and Richter., 2018). Decrease in calcium levels is due to a failure to transport calcium into the stratum granulosum. As a consequence, skin pH is increased and aged skin is more vulnerable to bacterial infection.

## Wound healing in diabetes

Diabetic foot ulcers (DFU) are the most common chronic wounds characterized by poor healing. Patients with diabetes mellitus have a 15%–25% lifetime risk of developing DFU, of which 40%–80% become so severely infected that they suffer from bone infection, leading to osteomyelitis. Wound healing disorders in patients with diabetes also occur due to a higher incidence of infectious complications, vascular changes at the level of microangiopathy and macroangiopathy, and in some cases repeated pressure on the wound increasing local ischemia. The issue of wound healing is a complex matter, so attention should be paid to the control of several parameters. The most important factor involves poor glycemic control (Dissanayake et al., 2020). Chronic decompensation of diabetes helps to develop ischemic lower limb disease, neuropathy, and other abnormalities that are modified not only at the systemic but also at the local level. Prolonged poor controlled diabetes leads to dysfunction of immune cells involved in repair processes and to the formation of late glycation products, which affect wound healing directly by reacting with some components of the healing process, or indirectly through diabetic neuropathy or angiopathy individual stages of the wound healing process (Fournet et al., 2018).

The early stages of wound healing are characterized by hypoxia, which induces the activation of hypoxia-inducible factor (HIF) -1α and stimulates the stimulation of vascular endothelial growth factor (VEGF-A). HIF is very important to promote the migration and proliferation of each cell type as well as the release of growth factors (Hong et al., 2014). HIF-1 is involved in many wound healing processes; such as cell migration, cell metabolism under hypoxic conditions, cell differentiation, cell growth factor release, cell survival, and synthesis of signal molecules throughout the healing process. Both overexpression of HIF-1, as well as HIF-1 deficiency, are associated with reduced adaptive responses to hypoxia during diabetic wound healing (Li et al., 2021). Overexpression of HIF-1 leads to an increased production of profibrotic factors associated with the overproduction of collagenous matrix (Kimura et al., 2008). On the other hand, HIF-1 deficiency and subsequent impaired response to hypoxic stimuli contribute to the formation of non-healing ulcers. Targeted wound healing therapy using regulators of HIF-1 production has many important aspects that can lead to tissue repair (Li et al., 2021). However, more preclinical and clinical studies are needed to validate the feasibility of treating diabetic wounds by manipulating HIF-1α activity.

In the damaged tissue, monocytes are activated, which become macrophages and they mediate phagocytosis as well as the production of growth factors such as platelet-derived growth factor (PDGF), tumor necrosis factor (TNF), and transforming growth factor (TGF-β). Growth factors influencing wound healing include PDGF, which increases macrophage migration and collagen synthesis, promotes granulation tissue formation and accelerates epithelialization, fibroblast growth factor (FGF) supporting angiogenesis and fibroblast proliferation, insulin-like growth factor (IGF-1) increasing fibroblast proliferation, collagen synthesis and epithelialization (Patel et al., 2019; Garoufalia et al., 2021). Decreased IGF-1 expression in diabetic individuals has been reported, which in turn has slowed the healing process (Garoufalia et al., 2021; Liu et al., 2021). Many (23 types) of FGF have been identified and divided into seven subfamilies. Recently, with the increasing research on the function of FGF, more and more studies are focused on FGF therapeutic approach. It has been applying FGF-1, FGF-2, FGF-4, FGF-7, FGF-21, and FGF-23 topically to DFU with good therapeutic effects (Liu et al., 2021).

The other three factors—TGF-beta, TNF-alpha, and IL-1 (interleukin-1) promote angiogenesis and collagen synthesis and therefore regulate epidermal stem cells in wound epithelialization (Xiao et al., 2020). The last factors influencing wound healing are GSF (granulocyte stimulating factor) and VEGF (vascular endothelial growth factor). The latter was significantly reduced during wound healing (by 50%) in an experiment performed on diabetic rats compared to healthy controls (Kurkipuro et al., 2022).

The anti-inflammatory effect of epidermal growth factor (EGF) has been intensively studied for more than 20 years. Many studies showed that the intralesional administration of EGF has emerged as an effective treatment for DFU (Mendoza-Mari et al., 2022). EGF injected into the ulcer matrix enhanced cell proliferation and migration, leading to peri- and intra-lesion infiltration. Therefore, it accelerates the healing of deep and complex ulcers, both ischemic and neuropathic, and reduces diabetes-related amputations (Berlanga et al., 2013). EGF helps diabetic wound healing, reaching responsive cells while avoiding the deleterious effect of proteases and the biofilm on the wound’s surface.

Gasotransmitter’s use was tested also in treatment of chronic diabetic wounds. Nitric oxide and NO-releasing compounds can significantly contribute to diabetic wound healing. Since in diabetes endogenous production of NO is affected, need for the topical supply of NO from exogenous sources is desirable (Takagi et al., 2022). Azelnipidine, a new dihydropyridine blocker of L-type calcium channels, increased wound fluid NO level, enhanced fibroblast proliferation and promotes angiogenesis, which participates to the acceleration of wound healing in type 1 diabetic rats (Bagheri et al., 2011). The NO-donors attached to patches or matrices to treat diabetic wounds are under development. Recently, preparation of anti-bacterial and nano-enzyme-containing hydrogel with inflammation-suppressing, ROS-scavenging, oxygen and nitric oxide-generating properties was published (Tu et al., 2022).

Insufficient intracellular H_2_S production in diabetes impairs angiogenic property and ischemic tissue injury, probably *via* interrupting the balance between pro- and anti-angiogenic factors (Cheng and Kishore, 2020). Exogenous donation of H_2_S by NaHS improved diabetic wound healing in ob/ob mice via promoting angiogenesis and attenuating inflammation (Zhao et al., 2017).

A meta-analysis was performed by Lin et al. (2022) focused on the association between vitamin D levels, respectively vitamin D hypovitaminosis, and wound healing in diabetic patients. Most significant association has been found between low vitamin D levels and foot ulcer wounds. Patients with foot ulcer wounds had significantly lower levels of vitamin D. Also, higher prevalence of vitamin D deficiency as well as higher prevalence of severe vitamin D deficiency was associated with higher incidence of foot ulcer wounds compared with non-diabetic non-ulcerated diabetic subjects. Some studies suggest that vitamin D supplementation in diabetic patients may have a positive effect on foot ulcer wound healing (Yammine et al., 2020; Halschou-Jensen et al., 2021; Kurian et al., 2021). Severe vitamin D deficiency is associated with elevated inflammatory cytokine concentrations in diabetic patients, particularly in those with foot infection (Tiwari et al., 2014). Negative correlation was observed between vitamin D and circulating concentrations of IL-1 beta and IL-6, but not for TNF-alpha and IFN-gamma. The question remains as to the mechanism of action of vitamin D in wound healing. Topically applied vitamin D in diabetic patients promotes corneal wound healing and nerve regeneration, reduces neutrophil infiltration and stimulates the transition of macrophages from M1 to M2, which is accompanied by suppression of excessive activation of the NLRP3 inflammasome (Wang et al., 2021b). Vitamin D downregulates the expression of MMP-1 and MMP-10 in keratinocytes from diabetic food ulcer cultivated *in vitro*. In contrast, increased expression of these genes was found in diabetic patients with diabetic food ulcer (Lopez-Lopez et al., 2014). MMP-1 breaks down the interstitial collagens I, II, and III, MMP-10 is intensively studied in connection with processes of metastasizing. The stem cells’ secreted bioactive molecules (the secretome) mediate paracrine and autocrine functions. Mesenchymal stromal cells (MSCs) are multipotent cells that reside in tissues and can give rise to bone, cartilage, adipocytes, or vascular smooth muscle cells (Laloze et al., 2021).

Meta-analyses of Jaluvka et al. (2020) reveals that cell therapy in peripheral arterial disease (PAD) treatment can prevent or delay foot amputation (Jaluvka et al., 2020). The wound healing process with stem cell therapy can be at least twice as shorter when compared with the standard conservative therapy. It can lead to improvement of perfusion and tissue oxygenation parameters in the wound, even more to pain regression. The available evidence-based medicine data showed that cells-based therapy is safe, associated with minimum complications or adverse events, and effective (Jaluvka et al., 2020). MSCs have been identified in tissues other than the bone marrow, including the umbilical cord, placenta, dental pulp, and adipose tissue (AD-MSCs). From the stem cell types, AD-MSCs have been intensively studied in terms of improving chronic wound healing (Ajit and Ambika Gopalankutty, 2021). The influence of ASC-secretome on cell types associated with the wound healing process can provide a basis for further and more targeted investigations that are useful for addressing the ways of accelerating chronic non-healing wound closure (Lombardi et al., 2019). A recent study showed that MSCs under TNF-α stimulation (MSC-CM-T) can release numerous trophic and survival molecules that have a promising prospect in wound healing acceleration in an animal model of wound healing. The topical gel of MSC-CM-T is more effective in accelerating wound closure healing through increasing platelet-derived growth factor (PDGF) levels and wound closure percentages and fibroblast density appearances in the skin defect animal models (Laloze et al., 2021; Putra et al., 2022).

## Conclusion

Wound healing is an extremely complex and complicated process that involves the integration of a variety of mechanisms at different time intervals along a timeline. Hemostasis, inflammation, proliferation, and remodelling are four stages of wound healing. Effect of gasotransmitters and modulation of calcium levels by calcium transport systems on individual phases of wound healing is summarized in Table 2. Development of new drugs targeting individual stages can provide a tool that can more effectively treat different types of wounds (e.g., diabetic wounds). Thus, new treatments based on precise knowledge of pathways activated in every stage can facilitate the process of wound healing. Calcium ions are known to play the crucial role in cell signalling. Several experimental studies have shown that calcium-releasing materials can significantly stimulate wound healing. They stimulate angiogenesis, collagen and extracellular matrix protein synthesis and overall tissue granulation. Polymeric composite dressings containing calcium-releasing nanoparticles are investigated as novel calcium-releasing systems that significantly accelerated wound healing in a diabetic (db/db) mouse model. Among new approaches, use of gasotransmitters provides an excellent tool for treatment, since they easily penetrate into the cells and they might stimulate proliferation (especially NO), they enhance vascularization and decrease period of treatment, especially in diabetic wounds.

**TABLE 2:** Involvement of gasotransmitters and calcium transport systems in wound healing.

Wound healing	Acute wounds	Chronic wounds
Inflammation	CO-antiinflammatory effect	H_2_S—attenuates inflammation
	NO- antimictobial effect	NO—suppresses inflammation, ROS scavenging
	H_2_S- antimicrobial effect	
	Ca^2+^ through TRPV—improves inflammatory wound healing	
Proliferation differentiation	CO-increases proliferation, differentiation	Ca^2+^ blocking by azelnipidine promotes fibroblast proliferation
	Ca^2+^ through RyR—promotes differentiation in keratinocytes	
	Ca^2+^ through AE2—promotes keratinocytemigration	
	Ca^2+^ through TRP—affects proliferation, differentiation	
Remodeling angiogenesis	H_2_S—increases blood perfusion around wounds	H_2_S—promotes angiogenesis
		NO through iNOS—enhancing angiogenesis
		Ca^2+^ blocking by azelnipidine promotes angiogenesis

AE2, bicarbonate transporter type 2; CO, carbon monooxide; H_2_S, hydrogen sulfide; iNOS, inducible NO synthase; NO, nitric oxide; ROS, reactive oxygen species; RYR, ryanodine receptors; TRP, transient receptor potential channel; TRPV, vanilloid transient receptor potential channel.

In summary, skin wounds often represent a burden to the patient, generally limiting his comfort. Therefore, effective healing, possibly without scars, represents a goal in dermatology. Development of new strategies of wound healing is based on current knowledge of modulated signaling pathways in the wound. Recently, modern treatments based on impregnation of hydrogels and nanoparticles with gasotransmitters, blockers of calcium transport, vitamins, etc. form a powerful tool for effective wound healing.

## Abbreviations

AE2, bicarbonate transporter type 2; CBS, cystathionine β-synthase; CSE, cystathionine γ-lyase; CRBP-1, cellular retinoid-binding protein 1; DFU, diabetic foot ulcers; ECM, extracellular matrix; EGF, epidermal growth factor; eNOS, endothelial NO synthase; FGF, fibroblast growth factor; HIF, hypoxia-inducible factor; HO-1, heme oxygenase-1; IGF, insulin-like growth factor; iNOS, inducible NO synthase; IP3Rs, inositol 1,4,5-trisphosphate receptors; MSCs, mesenchymal stromal cells; MST, 3-mercaptopyruvate-sulfurtransferase; nNOS, neuronal NO synthase; Nrf2, nuclear factor (erythroid-derived 2)-like 2; PAD, peripheral arterial disease; PDGF, platelet-derived growth factor; PPAR, peroxisome proliferator-activated receptors; RARs, retinoic acid receptors; ROS, reactive oxygen species; RXRs, retinoid X receptors; RyRs, ryanodine receptors; TGF, transforming growth factor; TNF, tumor necrosis factor; TRP, transient receptor potential channel; VEGF-A, vascular endothelial growth factor.
